# MRI of atherosclerosis and fatty liver disease in cholesterol fed rabbits

**DOI:** 10.1186/s12967-018-1587-3

**Published:** 2018-08-01

**Authors:** Erik Taylor, Nasi Huang, Jacob Bodde, Andrew Ellison, Ronald Killiany, Markus Michael Bachschmid, James Hamilton

**Affiliations:** 1 Department of Physiology and Biophysics, Boston University School of Medicine, 700 Albany Street, W302, Boston, MA 02118-2526 USA; 2 Vascular Biology Section, Whitaker Cardiovascular Institute, and Cardiovascular Proteomics Center, Boston University School of Medicine, Boston, MA USA; 3 Anatomy and Neurobiology, Boston University School of Medicine, Boston, MA USA

**Keywords:** Steatohepatitis, Liver fibrosis, Inflammation, Vulnerable atherosclerotic plaques, Magnetic resonance imaging and spectroscopy

## Abstract

**Background:**

The globally rising obesity epidemic is associated with a broad spectrum of diseases including atherosclerosis and non-alcoholic fatty liver (NAFL) disease. In the past, research focused on the vasculature or liver, but chronic systemic effects and inter-organ communication may promote the development of NAFL. Here, we investigated the impact of confined vascular endothelial injury, which produces highly inflamed aortic plaques that are susceptible to rupture, on the progression of NAFL in cholesterol fed rabbits.

**Methods:**

Aortic atherosclerotic inflammation (plaque Gd-enhancement), plaque size (vessel wall area), and composition, were measured with in vivo magnetic resonance imaging (MRI) in rabbits fed normal chow or a 1% cholesterol-enriched atherogenic diet. Liver fat was quantified with magnetic resonance spectroscopy (MRS) over 3 months. Blood biomarkers were monitored in the animals, with follow-up by histology.

**Results:**

Cholesterol-fed rabbits with and without injury developed hypercholesterolemia, NAFL, and atherosclerotic plaques in the aorta. Compared with rabbits fed cholesterol diet alone, rabbits with injury and cholesterol diets exhibited larger, and more highly inflamed plaques by MRI (P < 0.05) and aggravated liver steatosis by MRS (P < 0.05). Moreover, after sacrifice, damaged (ballooning) hepatocytes and extensive liver fibrosis were observed by histology. Elevated plasma gamma-glutamyl transferase (GGT; P = 0.014) and the ratio of liver enzymes aspartate and alanine aminotransferases (AST/ALT; P = 0.033) indicated the progression of steatosis to non-alcoholic steatohepatitis (NASH).

**Conclusions:**

Localized regions of highly inflamed aortic atherosclerotic plaques in cholesterol-fed rabbits may contribute to progression of fatty liver disease to NASH with fibrosis.

**Electronic supplementary material:**

The online version of this article (10.1186/s12967-018-1587-3) contains supplementary material, which is available to authorized users.

## Background

One in four Americans, including an increasing number of young adults, develops non-alcoholic fatty liver (NAFL) [1]. After viral hepatitis, NAFL ranks among the most common liver diseases in the USA and worldwide [1, 2]. The liver is a central organ of lipid metabolism and plasma lipoprotein synthesis. Genetic, environmental, and dietary factors can contribute to lipid accumulation in the liver, referred to as hepatic steatosis [2, 3]. In Western countries, fat- and carbohydrate-rich diets are associated with the development of obesity, insulin resistance, hypertension, and atherosclerosis [3]. An imbalance in hepatic lipid metabolism, deposition, or de novo synthesis promotes lipid buildup in hepatocytes, the initial stage of NAFL. While the course of simple steatosis is reversible and in most cases asymptomatic, the chronic excess of intracellular lipids induces lipotoxicity [4] and hepatocyte injury which in some individuals can progress to fibrosis, cirrhosis, [5] and finally hepatocellular carcinoma [2, 6]. Damaged hepatocytes release intracellular transaminases, plasma biomarkers of liver injury, and may undergo cell death. These inflammatory and pro-fibrotic processes in response to injury advance liver disease to non-alcoholic steatohepatitis (NASH). Limited effective pharmacological therapies exist currently, and thus NASH is considered as an irreversible stage of NAFL [5].

Histological grading of NAFL patient liver biopsies showed that NASH occurs in 10–30% of patients [1, 7]. While knowledge has grown regarding lifestyle and dietary impacts of liver disease from cohort studies [7, 8], the role of vascular inflammation and its potential systemic effects on liver disease has been challenging to establish mechanistically. In this study, we further explore our ongoing hypothesis that unresolved local inflammation can produce a chronic systemic inflammatory assault that affects other tissues and organs [9, 10]. Previously, we showed that oral inflammation with *Porphyromonas gingivalis* exposure greatly exacerbates aortic atherosclerosis in cholesterol fed rabbits. Here, we test the hypothesis that progressive atherosclerotic plaque inflammation can promote progression of NASH into a pro-inflammatory phenotype with fibrosis. NASH is associated with increased risk of thrombus formation and pro-coagulation factors as demonstrated by recent clinical cohort [11] and epidemiologic studies [12]. Inflamed vulnerable plaques prone to thrombosis release pro-coagulation factors and could also advance liver pathologies. A lack of comprehensive multi-organ studies has hindered the development of effective therapeutics for the liver, while advancing liver diseases may tie together cardiometabolic abnormalities and vascular disease. For example, general anti-inflammatory therapies may help to reduce chronic systemic inflammation. This observation is further substantiated by a recent trial in patients with a prior heart attack who were treated with a drug canakinumab (a monoclonal antibody that neutralized IL-1-beta) that led to a reduction in the risk of a second heart attack [13].

Diets may contain either pro or anti-inflammatory components that modulate the systemic inflammatory state [14, 15]. In particular, specific lipid species may exhibit pro-inflammatory [2, 4], neutral [16, 17], beneficial [10, 18], or resolving effects on the vasculature, liver, and other organs. Excessive cholesterol (CHOL) accumulation in the liver promotes CHOL crystal formation which may play a direct role in hepatic lipotoxicity [4, 19] and inflammation [20–22]. Lipidomic analyses of human NASH livers showed an increase in CHOL levels, but not in simple steatosis, while levels of free fatty acids were unchanged [23–25]. Several representative epidemiological studies demonstrated the association of total CHOL intake with an increased risk and severity of NAFL [15], cirrhosis, or liver cancer [26, 27].

This study investigates whether local vascular inflammation in the lipid-rich atherosclerotic plaques of cholesterol fed rabbits promotes chronic systemic effects and accelerate liver disease from NAFL to NASH. For this, we used a well-established rabbit model of human atherosclerosis that replicates histological features of both, stable and vulnerable plaques [early (types II and III) and advanced (types IV, Va, Vc, VI)] at the end of the 3 months protocol [28]. In vivo MRI of the atherosclerotic aorta has provided quantitative imaging features that are characteristic of vulnerable plaques and predictive of thrombosis [29, 30]. Injury of the aortic endothelium in combination with 1% CHOL feeding has produced highly inflamed atherosclerotic plaques [28, 31]. Severe NASH developed only in rabbits receiving both 1% CHOL diet and injury, in comparison to rabbits receiving either 1% CHOL diet or normal diet with injury alone.

## Methods

### Rabbit model of atherosclerosis

Male and female rabbits were fed normal chow and 1.0% CHOL containing chow diet for 3 months (Table 1 includes all of the study groups and abbreviations). For one study group (1% CHOL + injury; N = 5; two male and three female) the rabbits were prepared as previously described [28, 29, 32]. Briefly, advanced highly inflamed atherosclerotic lesions were induced with a 1% CHOL diet (TestDiet, Saint Louis, MO) in conjunction with endothelial cell injury via balloon catheter procedure under general anesthesia (acepromazine, 0.75 mg/kg IM; ketamine, 35 mg/kg IM; xylazine, 2.5 mg/kg IM). For the second study group, rabbits were fed 1% CHOL diet without injury (1% CHOL; N = 3; one male and two female) [10]. Control rabbits (normal diet; N = 3; female) were fed normal chow (LabDiet, Saint Louis, MO). The sham rabbit was injured by balloon catheter and fed a normal diet throughout the study (normal diet + injury; N = 1; female). Normal chow contained 16.5% protein, 41% carbohydrates, 22.5% fiber, 2% fat whereas the CHOL containing chow included 10,000 ppm cholesterol, 14.4% protein, 42.8% carbohydrates, 23.7% fiber, 2.4% fat by weight. The remaining mass consisted of moisture, ash, vitamins, and minerals. Animal chow was stored in a cool, dry location with refrigeration.

**Table 1:** The rabbit groups used in this study

Groups used in this study	Abbreviation^a^
Normal diet + no injury	Normal diet
Normal diet + endothelial injury	Sham rabbit
1% cholesterol diet + no injury	1% CHOL
1% cholesterol diet + endothelial injury	1% CHOL + injury

^a^Abbreviation denotes the abbreviated names used in this study

**Fig. 1:**
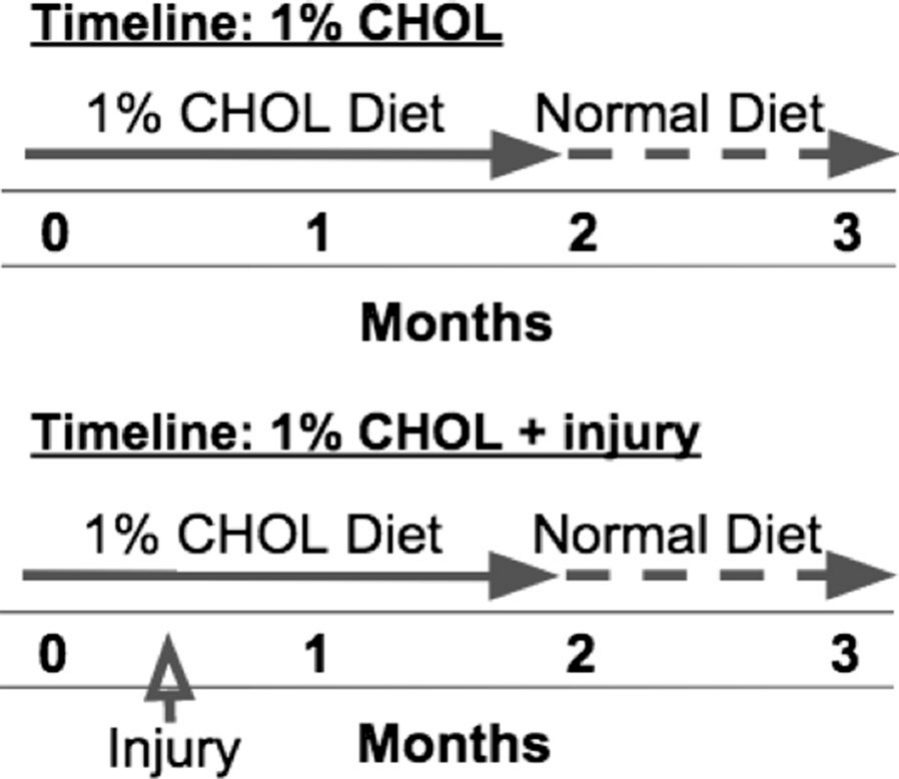
Timelines for the CHOL-fed rabbits with or without injury. The first timeline shown includes 2 months of 1% CHOL feeding followed by 1 month of normal diet. The second timeline adds endothelial injury carried out in the 1% CHOL + injury rabbit groups at 2 weeks. MRI in vivo was carried out at 2 months, followed by two repeat MRI sessions at 3 months to assess plaque rupture by pharmacologic triggering

We tested whether plaques disrupted to form a luminal thrombus in both 1% CHOL-fed groups with or without injury using our standard pharmacologic procedure [29, 31, 32]. Briefly, at the end of the 3 months protocol, intraperitoneal injections (two times, separated by 48 h) of coagulation cascade factor-X activating enzyme isolated from Russell’s Viper Venom (RVV-X 0.15 mg/kg IP; Enzyme Research, South Bend, IN) followed by histamine injection (0.02 mg/kg IV; Sigma Aldrich, St. Louis, Mo) after 30 min were used to trigger rupture of vulnerable plaques. We also performed balloon-catheter endothelial injury with pharmacologic triggering in a rabbit with no CHOL feeding as the sham group (normal diet + injury). The complete timeline for the CHOL-fed experimental groups included 8 weeks of 1% CHOL feeding followed by 4 weeks of normal chow feeding (Fig. 1).

### Serial magnetic resonance imaging (MRI) of aortic plaque and liver spectroscopy

Assessment of the aorta and liver in each rabbit was carried out for the detection of co-existing disease with in vivo MRI using a Philips Achieva 3T scanner with a 16 channel TR knee coil under general anesthesia and free breathing conditions. The MRI protocol was optimized based on our published protocols [29, 32] with modifications including T1-weighted (T1W) and T2-weighted (T2W) imaging substituted with non-gated 3D black-blood sequences repeated at the end of the second and third months. Our imaging protocol included additional experiments to quantify liver triglyceride content by voxel-guided 1H NMR spectroscopy (MRS) [33, 34]. Progression of aortic vascular disease was monitored with inclusion of intravenously injected gadolinium (0.5 ml of Magnevist, Bayer AG, Leverkusen, Germany) [32].

High field MRI (11.7T; Bruker Biospin Corporation, Billerica, MA) was used to study excised aortic segments rinsed in cold saline, fixed in 10% neutral buffered formalin, and then placed in MR-signal inert fomblin oil (Sigma Aldrich). The fomblin oil suppresses background signal, providing high signal-to-noise proportion during visualization of the tissue. Aorta segments were stored in an ice-water bath and allowed to equilibrate to room temperature prior to the ex vivo MRI protocol. All experiments were conducted with high resolution T1W imaging (28 μm^2^ in plane resolution with 500 μm slice thickness). Diffusion-weighted MRI (DW-MRI; 39 μm^2^ in plane resolution with 500 μm slice thickness) was used with a diffusion-weighting B value of 3000 s/mm^2^. The diffusion-weighting B value was selected and optimized based on the protocol developed by Qiao et al., for ex vivo visualization of cholesteryl esters in human and rabbit plaques [28, 35].

### MRI and MRS data analysis

From in vivo MRI measurements, we assessed vessel wall area (VWA) and plaque gadolinium (Gd) contrast enhancement. Selected segments representing regions with and without plaque rupture and normal vessel areas were compared. For Gd enhancement quantification, pre and post-contrast T1W images were used with a plaque defined as an area of hyperintensity present in at least three consecutive MRI slices (4–8 of these plaque regions were identified in each rabbit). For MRS measurements, the water peak was assigned to 4.7 ppm and lipid signal identified by characteristic peaks between 0.8 and 1.4 ppm [35]. Spectra from TG show intense resonances from the methylene (1.2 ppm) protons of fatty acyl chains [36]. From this MRS spectrum, the ratio of the methylene peak to the water peak was used, as is standard for measurement of liver fat [33, 34]. Spectra were averaged across eight readings at each of two sites (left and right liver) obtained during each imaging session. MATLAB software (MathWorks, Inc., Natick, MA) was used for calculation of the area under curve for the water and fat peaks. From ex vivo MRI, T1W imaging was used to quantify VWA. T1W is the standard scan to detect plaque heterogeneity. DW-MRI was used for ex vivo visualization to detect the presence of cholesteryl esters in the vessel wall [28, 35].

### Thrombus detection

At the end of the 3 months protocol (Fig. 1), thrombus formation was measured in ImageJ (NIH, Bethesda, MD) by comparison of carefully matched corresponding regions in the MR images (pre- and post-contrast T1W images) obtained before and after triggering.

### Blood biomarkers

Blood was collected prior to MRI scans (total volumes per draw of 2–4 ml) in heparin blood collection tubes for quantification of liver enzymes (U/l) and lipids (mg/dl). The biomarker analysis included measurements of the liver enzymes: aspartate aminotransferase (ALT), alanine aminotransferase (AST), and gamma-glutamyl transferase (GGT). The ratio of AST and ALT, also known as the De Ritis ratio [37], was used to assess liver damage in NASH [38]. Other biomarkers measured included: total plasma triglycerides, plasma free CHOL, and total protein (g/dl) concentrations. Blood lipoproteins (mg/dl) were measured and compared to normal diet fed rabbits, including high density lipoprotein (HDL), low density lipoprotein (LDL), and very-low density lipoprotein (VLDL). All blood biomarkers were measured by the Abbott Piccolo Xpress Analyzer (Abbott, Chicago, IL).

### Histology

Formalin fixed and paraffin embedded liver and aorta sections were stained with Masson’s Trichrome. Fibrotic areas stained blue were quantified after microscopic imaging and in multiple fields of view at the same magnification with ImageJ software using color threshold to quantify liver fibrosis. Lipid deposition in aortic frozen sections was visualized by Oil Red O.

### Statistical analysis

Statistical comparison was carried out on data derived from 1% CHOL + injury, 1% CHOL, or normal diet rabbit groups. In addition to comparisons between groups at study time points, baseline and longitudinal measurements were included where possible. Two-sample Student’s t-test for the mean difference per rabbit, one-sided or one sample t-test for comparison with baseline readings, and the F-test for the comparison of variance was carried out in Excel (Microsoft Corporation, Redmond, WA). Alternatively, data were analyzed with the R programming language and Rstudio (Boston, MA) for the analysis of variance (ANOVA) in the three groups, or the Wilcoxon rank sum test, a non-parametric alternative to the t-test, with visualization as dot plots.

## Results

### In vivo MRI of atherosclerosis and thrombosis

We previously demonstrated the application of non-invasive MRI to quantify the progression of individual atherosclerotic plaques in injured aortas of CHOL-fed rabbits. Plaques that exhibited thrombosis at 3 months were characterized by greatly increasing inflammation between the second and third months [32], even though the rabbits were fed normal chow for the last month. In the current study, we applied serial in vivo MRI to monitor atherosclerosis and MRS to quantify liver triglyceride in rabbits fed 1% CHOL with and without endothelial injury of the aorta and subsequently compared the livers in each group by histology.

**Fig. 2:**
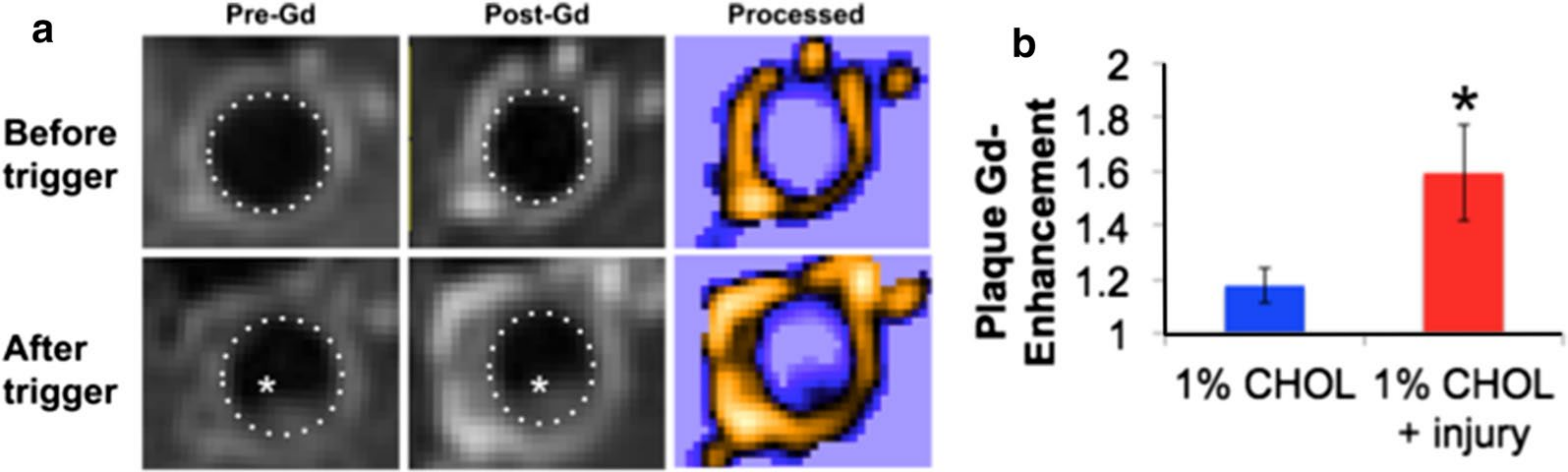
Plaque imaging and feature quantification in vivo with intravenous gadolinium (magnevist) in T1W MRI. **a** An example of plaque thrombosis by comparison of images of the aorta before and after pharmacologic triggering. The luminal thrombus is shown with a white asterisk in the images of endothelial-injured rabbits fed 1% CHOL. The plaque area is hyperintense with the lumen wall outlined (dotted line). Colorized and contrast enhanced images (labeled as “Processed”) further demonstrated thrombus boundaries and regions of enhanced gadolinium uptake into the plaque, characteristic of highly inflamed tissue. **b** Quantification of Gd uptake in the plaques measured at 3 months prior to triggering (n = 3 for 1% CHOL and n = 4 for 1% CHOL + injury). The data represent multiple plaques analyzed (4–5 plaques for 1% CHOL and 6–8 plaques per 1% CHOL + injury), with a plaque defined as an area of hyperintensity around the vessel wall, present in at least three consecutive MRI slices of the imaging volume. Gd uptake is higher for most plaques in the injury group as represented by the mean +/− standard error. Asterisk indicates significance with P < 0.05 analyzed with the Student’s t-test (pre-trigger). Enhanced Gd uptake is one of three metrics for predicting vulnerable plaque rupture, which also includes vessel wall area and outward remodeling [29, 32]

Consistent with our previous study using serial MRI [32], vulnerable plaques showed increasing VWA and Gd uptake in rabbits with injury before rupture and luminal thrombosis. In vivo MRI located advanced vulnerable plaques in the abdominal aorta that thrombosed after pharmacological triggering (Table 2). The intravenous Gd injection enhanced the differentiation between plaque and thrombus, as shown in Fig. 2a. Gd uptake was higher for most plaques in the 1% CHOL-fed rabbits with injury in the aorta compared to 1% CHOL-fed rabbits without injury (Fig. 2b). Enhanced Gd uptake is one of three metrics for predicting inflammation and vulnerable plaque rupture by MRI, which also includes vessel wall area and outward remodeling [29, 32]. In vivo MRI measurements in aortic plaques of non-injured 1% CHOL-fed and injured rabbits fed normal diets has not been previously performed. Our new MRI results demonstrate less inflammation in the aorta of non-injured 1% CHOL-fed rabbits and injured rabbits fed normal diets. Serial MRI showed the minimal occurrence of vulnerable plaques, including the Gd enhancement, as compared to the 1% CHOL + injury rabbit group.

**Table 2:** Plaque ruptures resulting in thrombus detected by in vivo MRI in CHOL-fed rabbits

Group	Thrombus per rabbit
1% CHOL	0.67
1% CHOL + injury	3.75^a^

^a^ Indicates significance tested with Student’s t-test when thrombus per rabbit was compared between the two groups

### Vascular features with ex vivo MRI and histology

**Fig. 3:**
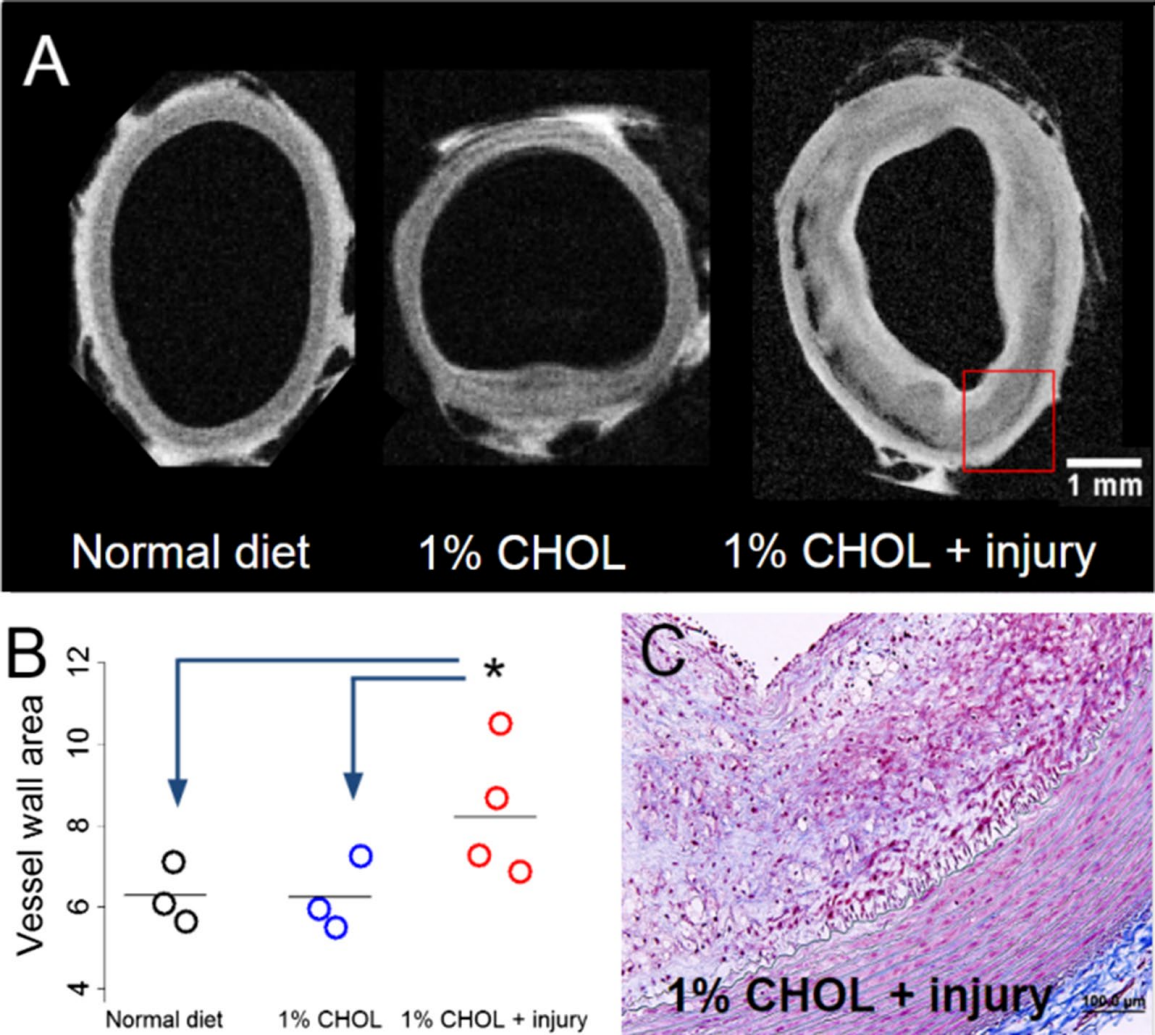
T1-weighted MRI of abdominal aortas ex vivo. **a** Representative images of vessel walls in normal diet fed, 1% CHOL, and 1% CHOL + injury rabbits. The thickened aortic vessel wall and increased wall area shown are characteristic of rabbits with 1% CHOL + injury after pharmacologic triggering. **b** Quantification of vessel wall area (mm^2^) as dot plots across rabbits and in multiple slices using ex vivo MRI. Each circle represents the average of eight slices for one rabbit with at least N = 3 in each category. The vertical lines represent the group mean value; P < 0.00005 was demonstrated by ANOVA, with similar results when comparing normal chow or 1% CHOL to the 1% CHOL + injury group by the Student’s t-test or Wilcoxon signed rank test. **c** Histological section from the 1% CHOL + injury aorta wall at 10× magnification from the ex vivo MRI image with Masson’s Trichrome staining (similar region as drawn in the red-box from (**a**). Scale bar size is 1 mm across images in (**a**) or 100 μm in (**c**)

After sacrifice, ex vivo MRI displayed higher vessel wall areas and greater compositional heterogeneity in the 1% CHOL + injury group compared to the 1% CHOL group and the normal diet fed rabbits (Fig. 3). We found relatively thin vessel walls in non-injured rabbits, very similar in morphology to 0.5% CHOL-fed rabbits in our previous studies using ex vivo MRI only [9, 10]. The heterogeneous composition in the 1% CHOL + injury group was revealed by high variation of signal intensities in the standard T1W image (Fig. 3a). The histology of the injury group confirmed the presence of a markedly thickened intima with inflammation (Fig. 3c).

**Fig. 4:**
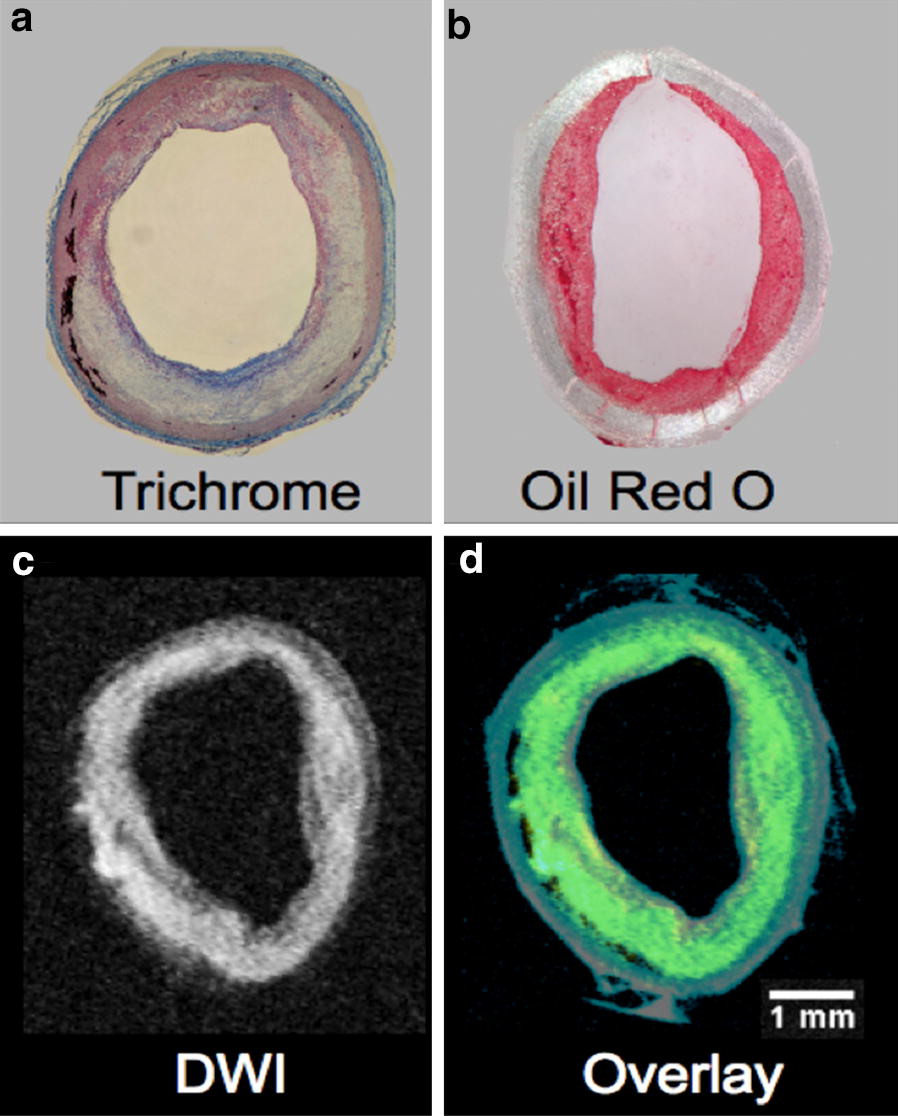
Displays of heterogeneous plaques with abundant lipids in 1% CHOL + injury rabbits by histology and ex vivo MRI. **a** Masson’s trichrome staining revealed massive heterogeneity with inflammation. **b** Abundant lipids in the luminal side of the vessel wall were visualized by Oil Red O staining. **c** Diffusion-weighted MRI (DW-MRI) confirmed substantial cholesteryl ester deposition in the luminal side of the vessel wall of 1% CHOL + injury rabbits [35]. **d** The DW-MRI sequence was overlaid on the standard T1W image showing all of the components of the vessel wall from 1% CHOL + injury rabbit. DW-MRI is depicted in yellow and T1W in blue. Overlapping regions result in the color green. Scale bar size inset is 1 mm across the images with equivalent scaling (**a**–**d**)

Abundant lipids were present in the vessel wall of the 1% CHOL fed rabbits with endothelial injury (Fig. 4). Oil Red O stain, a non-specific stain for lipids, was localized to the intimal region (Fig. 4b). The presence of the deposited lipid species was confirmed by ex vivo DW-MRI, (Fig. 4c) with specificity for liquid cholesteryl esters in the plaque [35]. Merged T1W with DW-MRI (in Fig. 4d) revealed the luminal side location of the lipid signal.

### Plasma biomarkers

A significant increase in lipids and liver enzymes (Table 3; 1% CHOL + injury and Additional file 1: Table S1; 1% CHOL**)** was observed in the blood of the CHOL-fed rabbits compared to rabbits fed normal chow. Free blood-cholesterol and triglycerides increased substantially with 1% CHOL feeding with injury. GGT, AST, and ALT were all increased by 165, 356, and 158%, respectively, although only the increase in GGT was significant. The ratio of AST to ALT increased from 0.63 to 1.11, and this effect was substantial (see Table 3 for complete details). A similar increase in free cholesterol was observed with 1% CHOL feeding without injury, (Additional file 1: Table S1) although increases in liver enzymes did not reach statistical significance for this group. The blood LDL + VLDL lipoprotein content was increased when compared between the normal diet and 1% CHOL diet fed rabbits with or without injury (results displayed in Additional file 1: Table S2).

**Table 3:** Change in liver enzymes in plasma with 1% CHOL diet and endothelial injury (N = 4)

Blood plasma biomarkers	Baseline	After 1% CHOL + injury	P-value
Free cholesterol (mg/dl)	48.5 ± 8.8	651.8 ± 167.5	0.018
Triglycerides (mg/dl)	44.3 ± 5.7	89.7 ± 15.9	0.021
GGT (U/l)	6.3 ± 0.5	10.3 ± 1.3	0.014
AST/ALT ratio	0.63 ± 0.12	1.11 ± 0.18	0.033
Total protein (g/dl)	4.9 ± 0.2	5.5 ± 0.4	NS

Readings from blood plasma were compared in 1% CHOL + injury rabbits to baseline readings. Baseline readings are defined as blood plasma samples obtained prior to initiation of 1% CHOL diet. Readings in the blood were obtained at 3 months before pharmacologic triggering. The P-value displayed was obtained from the Student’s t-test comparing the baseline and before trigger readings

### Liver triglyceride content by serial voxel-guided spectroscopy

**Fig. 5:**
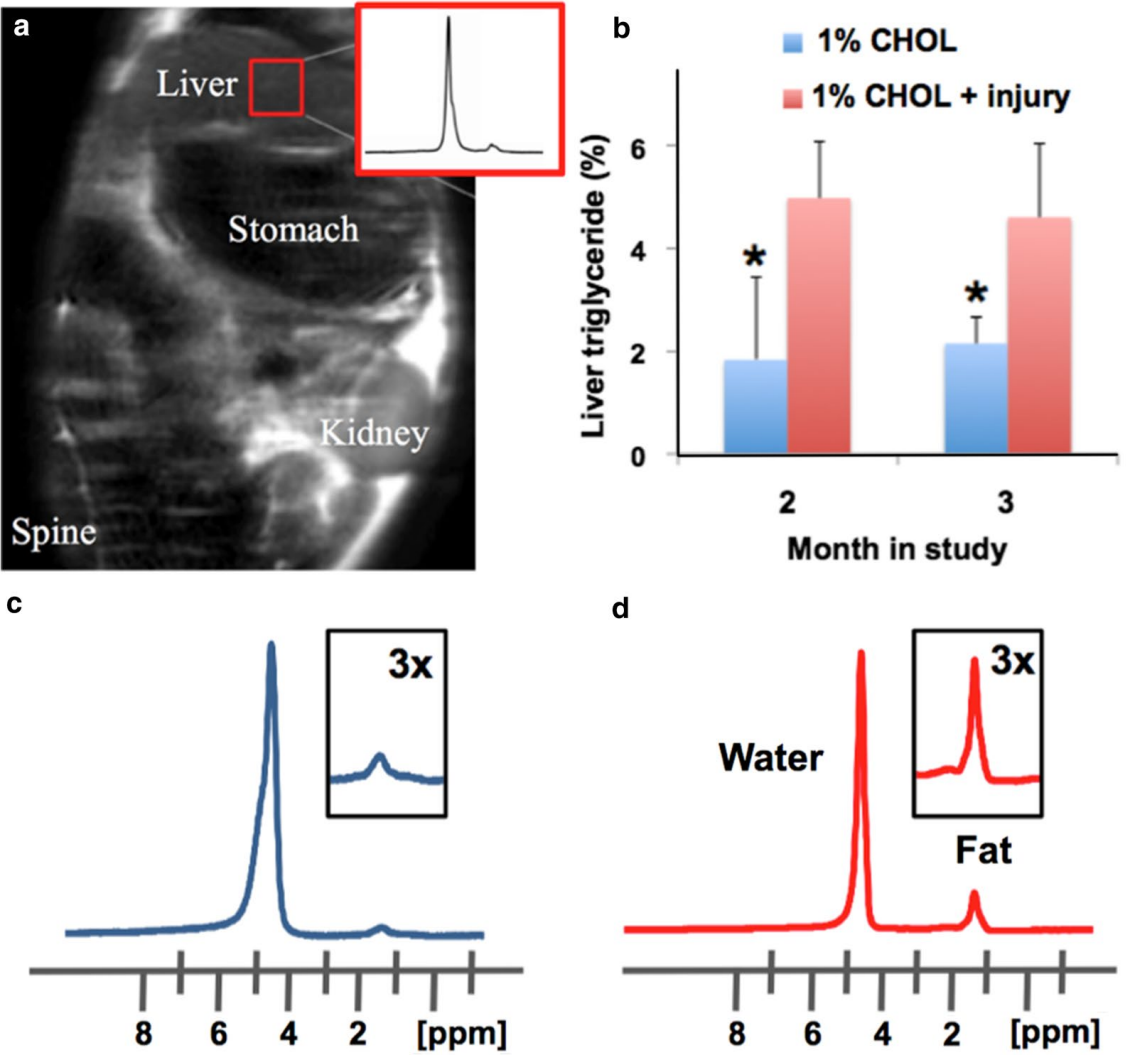
Lipid accumulation in the livers of CHOL-fed rabbits. **a** Liver triglycerides were quantified by MR spectroscopy (MRS) with selection of a voxel in images of the liver in vivo. The proton spectrum (inset in **a**) shows a major peak for water and a minor peak for the methylene groups of the fatty acyl chains in triglycerides. **b** MRS measurements of triglycerides in the livers of rabbits at 2 and 3 months were significantly higher in 1% CHOL-fed rabbits with endothelial injury versus 1% CHOL-fed rabbits with no injury (P < 0.05). Note that both groups were fed a normal chow diet for the last month (between 2 and 3 months). Representative MRS derived spectrums of liver water and fat in 1% CHOL-fed with no injury (**c**; 2.4% fat) and 1% CHOL + injury (**d**; 15.4% fat) at 3 months

We performed MRS of liver fat, which allows quantification of fat in the same region over time. Liver fat was quantified by the standard MRS method, as illustrated in Fig. 5. Liver triglycerides increased significantly at 2 and 3 months in 1% CHOL + injury compared to 1% CHOL rabbits. Accumulation of fat also resulted in brightening of the signal in T1W MRI of the liver.

### Liver histology with quantification of collagen fibrosis

**Fig. 6:**
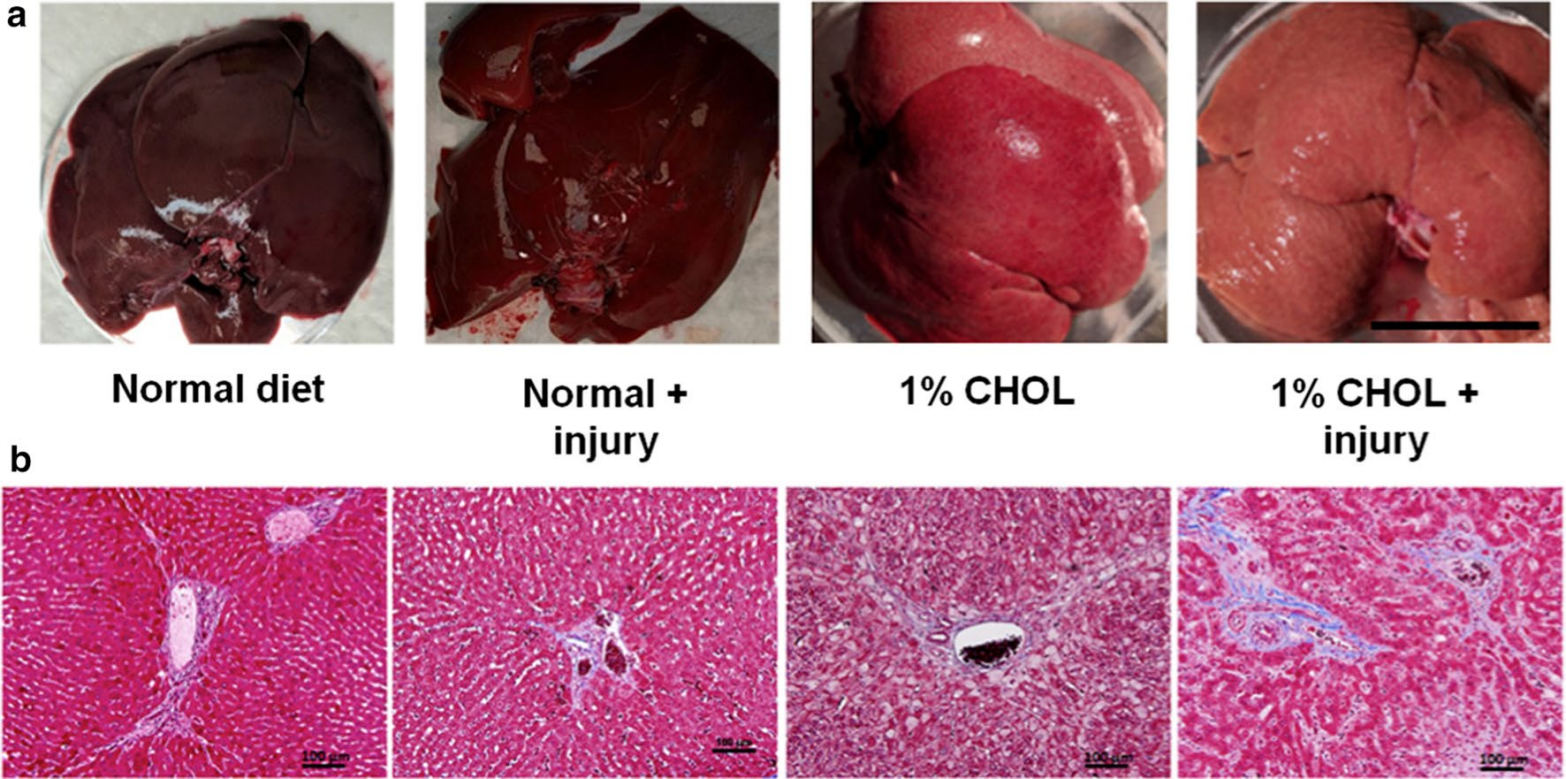
Lipid accumulation and fibrosis in the livers of CHOL-fed rabbits. **a** The livers of rabbits fed normal diets with or without injury appeared bright red and healthy. Substantial lipid accumulation in the liver caused visible pale or yellow color changes after 1% CHOL diet with or without endothelial injury. **b** Liver histology sections stained with Masson’s Trichrome revealed the presence of hepatocyte injury and fibrosis with CHOL feeding. The hepatocytes in livers of rabbits fed normal diets with or without injury appeared normal with low levels of fibrotic tissues present around the portal triads. Ballooning hepatocyte were observed in 1% CHOL fed rabbits with or without injury. The hepatocyte injury combined with marked fibrosis starting around the portal triads in the 1% CHOL + injury group rabbit livers indicated the presence of non-alcoholic steatohepatitis (NASH)

**Fig. 7:**
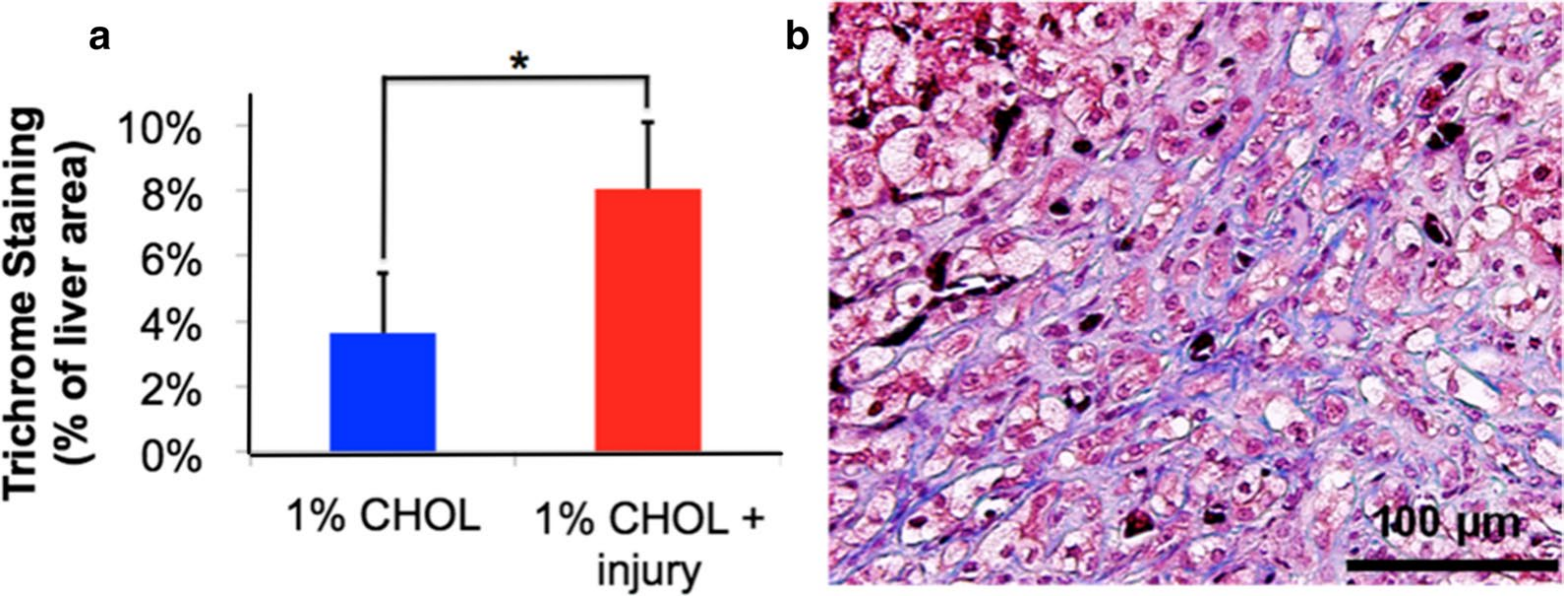
Quantification of fibrosis and fibrotic progression with cholesterol feeding and endothelial injury. Liver fibrosis was quantified in multiple microscopic imaging areas (**a**) with Masson’s Trichrome staining in the liver, demonstrating a significant 2.21 fold increase in fibrosis area with 1% CHOL + injury rabbits compared to 1% CHOL without injury (P < 0.005 by the Student’s t-test). **b** Non-injured rabbits fed 1% CHOL exhibited limited periportal fibrosis, whereas rabbits fed the same diet with endothelial injury showed marked progression of sinusoidal and periportal fibrosis as well as ballooning hepatocytes. The results shown are representative observations in multiple rabbits

When excised from the rabbits, a yellow or pale color change was apparent due to the presence of abundant lipids in CHOL-fed rabbits (Fig. 6a). Histology was then performed to determine the degree of liver inflammation and fibrosis (Fig. 6b). In rabbits fed normal diet with or without endothelial injury, normal liver histology was observed. The livers of rabbits fed 1% CHOL had ballooning hepatocytes, but with limited portal fibrosis. However, in rabbits fed 1% CHOL diet with endothelial injury, portal fibrosis extended into the liver tissue as shown in Fig. 7. The 1% CHOL + injury group had a two-fold increase in fibrosis area as compared to the CHOL-fed rabbits without injury.

## Discussion

The focus of this study was to compare liver disease in 1% CHOL fed rabbits with and without aortic injury and to address whether high vascular inflammation affected the liver. As shown by our in vivo MRI, ex vivo MRI, and histology of aortic atherosclerotic plaques, rabbits *without endothelial injury* developed smaller plaques with low levels of inflammation, whereas rabbits *with injury* developed more advanced plaques with high inflammation and a much higher frequency of disruption. We hypothesized that comparison of these groups with the same high CHOL diet (1%) would provide a better understanding of the impact of atherosclerotic inflammation that adversely affects other tissues such as the liver. Moreover, rabbits fed normal diets with or without injury did not have signs of progressive disease (plaques) in the aorta and lacked signs of liver steatosis or portal fibrosis.

Our rabbit model with CHOL feeding and injury is an established model of human atherosclerosis resembling most of the stages categorized by the American Heart Association (as noted in the *Introduction* and in [28]). Rabbits with 1% CHOL + injury also have numerous vulnerable plaques that after pharmacological triggering disrupt and form large thrombi (Fig. 2). Our in vivo results demonstrated greater Gd uptake in the 1% CHOL + injury group compared to the 1% CHOL without injury, which is a marker of active inflammation and neovascularization occurring in the vessel wall [29, 39]. The 1% CHOL rabbits had a smaller number of thrombi as compared to the 1% CHOL + injury group (Table 2). Similar results were previously observed by Abela et al. [31] using 1% CHOL-fed rabbits with endothelial injury compared to 1% CHOL-fed rabbits without injury, or injured rabbits fed normal diets. In our study, the 1% CHOL, normal diet, or normal diet + injury groups had relatively thin vessel walls compared to the 1% CHOL + injury group. Moreover, histological inflammation, compositional heterogeneity, and lipid contents were substantial in the vessel walls of the 1% CHOL + injury group (Figs. 3 and 4).

In addition to representing the human plaques, rabbits have high plasma LDL, VLDL, and other important plasma components, such as CRP [40–42]. Plasma free CHOL and triglycerides were elevated in 1% CHOL + injury rabbits (Table 3), as were all the lipoprotein subfractions HDL, LDL, VLDL (Additional file 1: Table S2). The AST to ALT ratio, heightened in the 1% CHOL + injury rabbits (Table 3), is a clinically valuable marker of liver dysfunction [37, 38]. An increase in the AST to ALT ratio was observed in the 1% CHOL fed rabbits without injury group (Additional file 1: Table S1), but to a lesser degree that did not reach statistical significance. A study of 70 patients with NASH revealed mean AST to ALT ratios of 0.7, 0.9, and 1.4 for subjects with no fibrosis, mild fibrosis, or cirrhosis, respectively [38]. In a larger clinical cohort, patients with no liver fibrosis and diabetes had a mean AST to ALT ratio of 0.78, and this was elevated to 0.98 (n = 204) in diabetic patients with advanced fibrosis (n = 142). Advanced fibrosis in the study was defined via histological diagnosis of either bridging fibrosis or cirrhosis [43]. GGT is also relevant to liver function, is responsible for the extracellular catabolism of the antioxidant glutathione, and is suspected to be important beyond liver damage in chronic subclinical inflammation and systemic oxidative demand [44–46].

Our report is the first study to our knowledge tracking advanced stage plaques that are highly inflamed in relation to liver disease in rabbits. The 1% CHOL + injury rabbits had significantly increased triglyceride deposition in the liver as observed by in vivo MRS at 2 and 3 months compared to 1% CHOL non-injured rabbits at the same time points (Fig. 5). Note that both groups were fed a normal chow diet for the last month (our standard protocol [28, 29, 32] and shown in our timeline), and the liver triglyceride in each group did not increase between 2 and 3 months. There were insignificant gains in weight over the entire 3 month time period in each cholesterol fed group. Therefore, the accumulation of body fat is not a contributor to the differences in liver pathologies between the two cholesterol fed groups of rabbits. We also found that endothelial injury alone without cholesterol feeding does not induce visible histologic liver steatosis with inflammation (as shown in Fig. 6).

Taken together, above results strongly suggest that vascular inflammation from lipid-rich plaques in the setting of the high cholesterol diet is a major contributor to the liver pathology. After completion of the MRI and MRS studies, the color of the CHOL-fed rabbit’s livers changed from normal deep red to pale or yellowish, most notable for the 1% CHOL + injury group (Fig. 6a). The most remarkable and physiologically substantial differentiation of the livers was revealed by histology, which showed propagating fibrosis in the 1% CHOL + injury rabbits, as compared to the other study groups (Fig. 6b).

Some fibrosis was noted in the 1% CHOL-fed rabbits without injury. In rabbits fed 1% CHOL alone, a mean of 3.65% liver fibrosis by area was found compared to 8.06% liver fibrosis by area in the 1% CHOL + injury rabbits (Fig. 7a). The propagation to bridging fibrosis far beyond the near-boundaries of the liver portal triad (bile duct, hepatic portal vein, and hepatic artery) was observed only in the 1% CHOL + injury rabbits (Fig. 7b). These findings suggests that liver fibrosis is initiated around the blood vessels, and with a more severe disease, collagen fibrosis increased to propagate into the hepatic sinusoids. The three main structure of the liver, the portal vein, artery, and bile duct, are surrounded by loose myofibroblasts and the first layer of hepatic and non-parenchymal cells. These portal myofibroblasts (and not hepatic stellate cells) are hypothesized to be the point of portal fibrosis in the early stages of cholestatic fibrosis [47].

Several localized diseases that are characterized by unresolved inflammation have now been linked to cardiovascular disease (CVD). Previous studies in rabbits have demonstrated that periodontitis greatly promotes atherosclerotic plaque inflammatory processes [9]. High circulating inflammatory mediators have been postulated to contribute to “vulnerable blood”, a systemic characteristic of high-risk for cardiovascular events in humans [9, 10, and references therein]. One study has also linked periodontitis to liver inflammation [48]. Psoriasis is another example of a link between discrete sites of inflammatory pathology that promotes vascular inflammation and early atherosclerosis. Psoriasis becomes a systemic inflammatory disease because of the failure to resolve localized inflammation and the secretion of high levels of neutrophils and inflammatory mediators into the blood [49]. Our new evidence from CHOL-fed rabbits *without* periodontitis or psoriasis shows that discrete pathological regions of lipid-rich inflamed pro-thrombotic plaques contribute to the progression of liver disease to advanced stages with high inflammation and fibrosis.

## Conclusions

This study has demonstrated that progressive atherosclerotic vascular inflammation is associated with worsening liver disease in rabbits. The liver disease appears to synergized between diet, aortic injury, and atherosclerotic inflammation, whereby the injury coupled with local plaque inflammation drives disease progression in both areas. These observations support the emerging broad hypothesis that unresolved inflammation may impart systemic effects. These findings provide a basis for detailed investigation of the mechanisms and molecules released from the plaques that can lead to inflammation and fibrosis in the liver. An important test of our overall hypothesis will be to assess the impact of pro-resolving mediators of inflammation, including lipoxins and resolvins, molecules that have been shown to be potent in treating or possibly preventing many inflammation-associated diseases [10].

## Additional file

**Additional file 1: Table S1.** Change in liver enzymes in plasma with 1% CHOL diet (N = 3). **Table S2.** Blood-lipoproteins in rabbits fed 1% CHOL diet with and without injury compared to rabbits fed a normal diet (N = 3 for normal diet, N = 3 for 1% CHOL diet, and N = 4 for 1% CHOL diet + injury).

## Abbreviations

NAFL: non-alcoholic fatty liver
MRI: magnetic resonance imaging
MRS: magnetic resonance spectroscopy
GGT: gamma-glutamyl transferase
AST: aspartate aminotransferases
ALT: alanine aminotransferases
NASH: non-alcoholic steatohepatitis
CHOL: cholesterol
RVV-X: Russell’s Viper Venom
T1W: T1-weighted
T2W: T2-weighted
DW-MRI: diffusion-weighted MRI
VWA: vessel wall area
Gd: gadolinium
HDL: lipoprotein
LDL: low density lipoprotein
VLDL: very-low density lipoprotein
ANOVA: analysis of variance
CVD: cardiovascular disease

## References

[1] Williams CD, Stengel J, Asike MI, Torres DM, Shaw J, Contreras M (2011). Prevalence of nonalcoholic fatty liver disease and nonalcoholic steatohepatitis among a largely middle-aged population utilizing ultrasound and liver biopsy: a prospective study. Gastroenterology.

[2] Ioannou GN (2016). The role of cholesterol in the pathogenesis of NASH. Trends Endocrinol Metab.

[3] Wree A, Broderick L, Canbay A, Hoffman HM, Feldstein AE (2013). From NAFLD to NASH to cirrhosis—new insights into disease mechanisms. Nat Rev Gastroenterol Hepatol.

[4] Neuschwander-Tetri BA (2010). Hepatic lipotoxicity and the pathogenesis of nonalcoholic steatohepatitis: the central role of nontriglyceride fatty acid metabolites. Hepatology.

[5] Cohen JC, Horton JD, Hobbs HH (2011). Human fatty liver disease: old questions and new insights. Science.

[6] Beste LA, Leipertz SL, Green PK, Dominitz JA, Ross D, Ioannou GN (2015). Trends in burden of cirrhosis and hepatocellular carcinoma by underlying liver disease in US veterans, 2001–2013. Gastroenterology.

[7] Matteoni CA, Younossi ZM, Gramlich T, Boparai N, Liu YC, McCullough AJ (1999). Nonalcoholic fatty liver disease: a spectrum of clinical and pathological severity. Gastroenterology.

[8] Clark JM (2006). The epidemiology of nonalcoholic fatty liver disease in adults. J Clin Gastroenterol.

[9] Hasturk H, Abdallah R, Kantarci A, Nguyen D, Giordano N, Hamilton J (2015). Resolvin E1 (RvE1) attenuates atherosclerotic plaque formation in diet and inflammation-induced atherogenesis. Arterioscler Thromb Vasc Biol.

[10] Hamilton JA, Hasturk H, Kantarci A, Serhan CN, Van Dyke T (2017). Atherosclerosis, periodontal disease, and treatment with resolvins. Curr Atheroscler Rep.

[11] Verrijken A, Francque S, Mertens I, Prawitt J, Caron S, Hubens G (2014). Prothrombotic factors in histologically proven nonalcoholic fatty liver disease and nonalcoholic steatohepatitis. Hepatology.

[12] Stine JG, Shah NL, Argo CK, Pelletier SJ, Caldwell SH, Northup PG (2015). Increased risk of portal vein thrombosis in patients with cirrhosis due to nonalcoholic steatohepatitis. Liver Transpl.

[13] Ridker PM, MacFadyen JG, Thuren T, Everett BM, Libby P, Glynn RJ (2017). Effect of interleukin-1β inhibition with canakinumab on incident lung cancer in patients with atherosclerosis: exploratory results from a randomised, double-blind, placebo-controlled trial. Lancet.

[14] Tabung FK, Liu L, Wang W, Fung TT, Wu K, Smith-Warner SA (2018). Association of dietary inflammatory potential with colorectal cancer risk in men and women. JAMA Oncol.

[15] Ioannou GN, Morrow OB, Connole ML, Lee SP (2009). Association between dietary nutrient composition and the incidence of cirrhosis or liver cancer in the United States population. Hepatology.

[16] Yamaguchi K, Yang L, McCall S, Huang J, Yu XX, Pandey SK (2007). Inhibiting triglyceride synthesis improves hepatic steatosis but exacerbates liver damage and fibrosis in obese mice with nonalcoholic steatohepatitis. Hepatology.

[17] Wouters K, van Bilsen M, van Gorp PJ, Bieghs V (2010). Intrahepatic cholesterol influences progression, inhibition and reversal of non-alcoholic steatohepatitis in hyperlipidemic mice. FEBS.

[18] Scorletti E, Byrne CD (2013). Omega-3 fatty acids, hepatic lipid metabolism, and nonalcoholic fatty liver disease. Annu Rev Nutr.

[19] Tabas I (2002). Consequences of cellular cholesterol accumulation: basic concepts and physiological implications. J Clin Invest.

[20] Musso G, Gambino R, Cassader M (2013). Cholesterol metabolism and the pathogenesis of non-alcoholic steatohepatitis. Prog Lipid Res.

[21] Arguello G, Balboa E, Arrese M, Zanlungo S (2015). Recent insights on the role of cholesterol in non-alcoholic fatty liver disease. Biochim Biophys Acta.

[22] Hendrikx T, Walenbergh S, Hofker MH (2014). Lysosomal cholesterol accumulation: driver on the road to inflammation during atherosclerosis and non-alcoholic steatohepatitis. Obesity.

[23] Min H-K, Kapoor A, Fuchs M, Mirshahi F, Zhou H, Maher J (2012). Increased hepatic synthesis and dysregulation of cholesterol metabolism is associated with the severity of nonalcoholic fatty liver disease. Cell Metab.

[24] Caballero F, Fernández A, De Lacy AM, Fernández-Checa JC, Caballería J, García-Ruiz C (2009). Enhanced free cholesterol, SREBP-2 and StAR expression in human NASH. J Hepatol.

[25] Puri P, Baillie RA, Wiest MM, Mirshahi F, Choudhury J, Cheung O (2007). A lipidomic analysis of nonalcoholic fatty liver disease. Hepatology.

[26] Musso G, Gambino R, De Michieli F, Cassader M, Rizzetto M, Durazzo M (2003). Dietary habits and their relations to insulin resistance and postprandial lipemia in nonalcoholic steatohepatitis. Hepatology.

[27] Yasutake K, Nakamuta M, Shima Y, Ohyama A, Masuda K, Haruta N (2009). Nutritional investigation of non-obese patients with non-alcoholic fatty liver disease: the significance of dietary cholesterol. Scand J Gastroenterol.

[28] Phinikaridou A, Hallock KJ, Qiao Y, Hamilton JA (2009). A robust rabbit model of human atherosclerosis and atherothrombosis. J Lipid Res.

[29] Phinikaridou A, Ruberg FL, Hallock KJ, Qiao Y, Hua N, Viereck JC (2010). In vivo detection of vulnerable atherosclerotic plaque by magnetic resonance imaging in a rabbit model. Circ Cardiovasc Imaging.

[30] Phinikaridou A, Hua N, Pham T, Hamilton JA (2013). Regions of low endothelial shear stress colocalize with positive vascular remodeling and atherosclerotic plaque disruption: an in vivo magnetic resonance imaging study. Circ Cardiovasc Imaging.

[31] Abela GS, Picon PD, Friedl SE, Gebara OC, Miyamoto A, Federman M (1995). Triggering of plaque disruption and arterial thrombosis in an atherosclerotic rabbit model. Circulation.

[32] Pham TA, Hua N, Phinikaridou A, Killiany R, Hamilton J (2016). Early in vivo discrimination of vulnerable atherosclerotic plaques that disrupt: a serial MRI study. Atherosclerosis.

[33] Lee SS, Park SH (2014). Radiologic evaluation of nonalcoholic fatty liver disease. World J Gastroenterol.

[34] Vanhamme L, van den Boogaart A, Van Huffel S (1997). Improved method for accurate and efficient quantification of MRS data with use of prior knowledge. J Magn Reson.

[35] Qiao Y, Ronen I, Viereck J, Ruberg FL, Hamilton JA (2007). Identification of atherosclerotic lipid deposits by diffusion-weighted imaging. Arterioscler Thromb Vasc Biol.

[36] Ruberg FL, Viereck J, Phinikaridou A, Qiao Y, Loscalzo J, Hamilton JA (2006). Identification of cholesteryl esters in human carotid atherosclerosis by ex vivo image-guided proton MRS. J Lipid Res.

[37] De Ritis F, Coltorti M, Giusti G (2006). An enzymic test for the diagnosis of viral hepatitis: the transaminase serum activities. 1957. Clin Chim Acta.

[38] Sorbi D, Boynton J, Lindor KD (1999). The ratio of aspartate aminotransferase to alanine aminotransferase: potential value in differentiating nonalcoholic steatohepatitis from alcoholic liver disease. Am J Gastroenterol.

[39] Kerwin WS (2010). Noninvasive imaging of plaque inflammation. JACC Cardiovasc Imaging.

[40] Okamoto H, Yonemori F, Wakitani K, Minowa T, Maeda K, Shinkai H (2000). A cholesteryl ester transfer protein inhibitor attenuates atherosclerosis in rabbits. Nature.

[41] Barter PJ, Brewer HB, Chapman MJ, Hennekens CH, Rader DJ, Tall AR (2003). Cholesteryl ester transfer protein: a novel target for raising HDL and inhibiting atherosclerosis. Arterioscler Thromb Vasc Biol.

[42] Fan J, Kitajima S, Watanabe T, Xu J, Zhang J, Liu E (2015). Rabbit models for the study of human atherosclerosis: from pathophysiological mechanisms to translational medicine. Pharmacol Ther.

[43] Bazick J, Donithan M, Neuschwander-Tetri BA, Kleiner D, Brunt EM, Wilson L (2015). Clinical model for NASH and advanced fibrosis in adult patients with diabetes and NAFLD: guidelines for referral in NAFLD. Diabetes Care.

[44] Melvin JC, Rodrigues C, Holmberg L, Garmo H, Hammar N, Jungner I (2012). Gamma-glutamyl transferase and C-reactive protein as alternative markers of metabolic abnormalities and their associated comorbidities: a prospective cohort study. Int J Mol Epidemiol Genet.

[45] Ali SS, Oni ET, Blaha MJ, Veledar E, Feiz HR, Feldman T (2016). Elevated gamma-glutamyl transferase is associated with subclinical inflammation independent of cardiometabolic risk factors in an asymptomatic population: a cross-sectional study. Nutr Metab.

[46] Bradley RD, Fitzpatrick AL, Jacobs DR, Lee D-H, Swords Jenny N, Herrington D (2014). Associations between γ-glutamyltransferase (GGT) and biomarkers of atherosclerosis: the multi-ethnic study of atherosclerosis (MESA). Atherosclerosis.

[47] Ramadori G, Saile B (2004). Portal tract fibrogenesis in the liver. Lab Invest.

[48] Goguet-Surmenian E, Hasturk H, Kantarci A, Andry C, Serhan CN, Van Dyke TJ (2009). Preclinical hepatotoxicity of lipid mediators in rabbits. J Dent Res.

[49] Sanda GE, Belur AD, Teague HL, Mehta NN (2017). Emerging associations between neutrophils, atherosclerosis, and psoriasis. Curr Atheroscler Rep.

