# Supplementary material for: MRI of atherosclerosis and fatty liver disease in cholesterol fed rabbits
Source: J Transl Med. 2018 Aug 1;16:215. doi: 10.1186/s12967-018-1587-3 (PMC6071403; doi:10.1186/s12967-018-1587-3)
Supplement: Supplementary file 1 — Additional file 1: Table S1. Change in liver enzymes in plasma with 1% CHOL diet (N = 3). Table S2. Blood-lipoproteins in rabbits fed 1% CHOL diet with and without injury compared to rabbits fed a normal diet (N = 3 for normal diet, N = 3 for 1% CHOL diet, and N = 4 for 1% CHOL diet + injury). [file 12967_2018_1587_MOESM1_ESM.docx]

| **Table S1.** **Change in liver enzymes in plasma with 1% CHOL diet (N=3).** | | | |
| --- | --- | --- | --- |
| **Blood Plasma Biomarkers** | **Baseline** | **After 1% CHOL** | **P-value** |
| Free Cholesterol (mg/dL) | 33.7 ± 19.4 | 659.7 ± 380.9 | 0.023 |
| Triglycerides (mg/dL) | 48.3 ± 27.9 | 112.3 ± 64.9 | NS |
| GGT (U/L) | 8.7 ± 5.0 | 12.7 ±7.3 | NS |
| AST/ALT ratio | 0.73 ± 0.42 | 1.0 ± 0.58 | NS |
| Total Protein (g/dL) | 4.9 ± 2.9 | 5.0 ± 2.9 | NS |
| Readings from blood plasma were compared in 1% CHOL rabbits without injury to baseline readings. Baseline readings are defined as blood plasma samples obtained prior to initiation of 1% CHOL diet. Readings in blood were also obtained at three months before pharmacologic triggering. The P-value displayed was obtained from the student’s t-test comparing the baseline and before trigger readings. | | | |

| **Table S2. Blood-lipoproteins in rabbits fed 1% CHOL diet with and without injury compared to rabbits fed a normal diet (N=3 for normal diet, N=3 for 1% CHOL diet, and N=4 for 1% CHOL diet + injury).** | | | | | |
| --- | --- | --- | --- | --- | --- |
| **Blood-lipoprotein** | **Normal Diet** | **1% CHOL** | **1% CHOL**  **P-value compared to Normal Diet** | **1% CHOL + Injury** | **1% CHOL + Injury: P-value compared to Normal Diet** |
| HDL (mg/dL) | 17.0 ± 0.9 | 32.3 ± 17.5 | NS | 50.1 ± 12.5 | 0.038 |
| LDL + VLDL (mg/dL) | 16.8 ± 1.5 | 426.0 ± 130.6 | 0.013 | 250.0 ± 28.3 | 0.0002 |
| All readings are from three months pre-trigger or prior to sacrifice in normal rabbits. The P-value displayed was obtained from the student’s t-test comparing normal diet to the 1% CHOL +/- injury rabbits. | | | | | |
